# Lipid-Lowering Effects of *Pediococcus acidilactici* M76 Isolated from Korean Traditional Makgeolli in High Fat Diet-Induced Obese Mice

**DOI:** 10.3390/nu6031016

**Published:** 2014-03-07

**Authors:** Yeon-Jeong Moon, Sang-Ho Baik, Youn-Soo Cha

**Affiliations:** 1 Department of Food Science and Human Nutrition, College of Human Ecology, Chonbuk National University, 567 Baekje-daero, Deokjin-gu, Jeonju-si, Jeollabuk-do 561-756, South Korea; E-Mails: biomoon@jbnu.ac.kr (Y.-J.M.); baiksh@jbnu.ac.kr (S.-H.B.); 2 Jeonju Makgeolli Research Center, Chonbuk National University, 567 Baekje-daero, Deokjin-gu, Jeonju-si, Jeollabuk-do 561-756, South Korea

**Keywords:** *Pediococcus acidilactici*, obesity, lipid metabolism

## Abstract

The effect of *Pediococcus acidilactici* M76 (lactic acid bacteria) isolated from makgeolli on mice fed a high fat diet was investigated to clarify the lipid lowering function. C57BL/6J male mice were randomly divided into a normal diet (ND) group, high fat diet (HD) group, HD plus *Pediococcus acidilactici* DSM 20284 reference strain (PR) group, and HD plus *Pediococcus acidilactici* M76 strain (PA) groups. The lyophilized PA and PR strain were dissolved in distilled water at a final concentration of 1.25 × 10^9^ cfu/mL and was given orally to animals at a dose of 4 mL/kg body weight for 12 weeks. The PA group had a lower final body weight, adipose tissue weight, and lipid profile than those in the HD group. Additionally, level of ACC, FAS and PPAR-γ, a key lipid synthesis enzyme, was markedly suppressed in the PA compared to those in the HD group. These data suggest that *P*. *acidilactici* M76 may exert a lipid-lowering effect in high fat diet- induced obese mice.

## 1. Introduction

Obesity is a medical condition characterized by accumulation of excess body fat. In Asia, increased consumption of energy dense food over the past decade has occurred both in urban and rural populations [1]. The majority of obese persons who develop cardiovascular disease typically have an involving elevated lipid levels and lipid signaling, inflammatory responses, insulin resistance, and adipokines [2,3]. In addition, medical costs for the obesity related diseases have increased from day to day, and the effort to decrease the percentage of body fat has become a worldwide interest. Thus, the developments of functional foods that emphasize decreased obesity are particularly important.

Korean rice wine has long been brewed traditionally using only nuruk, rice, and water. Rice wines contain 15%–18% alcohol before dilution. But, makgeolli is a low-alcohol beverage with an alcohol content of 6%–10%. Makgeolli contains essential amino acids, proteins, sugars, live yeast, and lactic acid bacteria (LAB) because the fermented product remains unfiltered. Thus, it has unique nutritional characteristics [4,5]. The major LAB identified in makgeolli is *Lactobacillus* sp. [6]. The presence ofother LAB such as *Leuconostoc*, *Pediococcus*, and *Enterococcus* sp. [7] depends on storage temperature and time [8,9]. 

LAB are major representatives of probiotics, which have been defined by the World Health Organization (WHO) as live micro-organisms which when administered in adequate amounts confer a health benefit on the host [10]. LAB used in various fermented food have therapeutic effects on human health, and their consumption has resulted in improvements of hepatic disease, allergies, hypertension, cancer, blood cholesterol and hyperlipidaemia [11,12]. The *Lactobacillus* sp. supplemented diet significantly delayed the onset of glucose intolerance in high fructose-induced diabetic rats, indicating a lower risk of diabetes and its complications [13]. Kadooka *et al*. suggested that *Lactobacillus gasseri* SBT 2055 reduces abdominal adiposity and body weight in adults with obese tendency [14]. 

Makgeolli inhibits growth of cancer cells [15] and has anti-complementary effects [16], antioxidant activity [17,18], anti-inflammatory effect [19]. Despite the importance of the nutritional effects of makgeolli, no study has been investigated how LAB from makgeolli affects lipid metabolism. Therefore, in this study, we investigated the influence of PA (LAB isolated from makgeolli) administration on C57BL/6J mice fed a high fat diet by examining changes in serum and liver lipid profiles and changes in hepatic mRNA levels of enzymes involved in lipid metabolism.

## 2. Experimental Section

### 2.1. Preparation of LAB Test Samples

PA, isolated from makgeolli, was used throughout this study (patent no. KACC91683P) [20]. *P*. *acidilactici* DSM 20284 (PR) was obtained as a reference strain from the Korean Agricultural Culture Collection (Suwon, Korea). Each strain was cultured in a 1000 mL flask containing 200 mL MRS (Difco, Detroit, MI, USA) broth on a rotary shaker incubator at 150 rpm for 24 h at 37 °C. After the incubation, the bacterial pellet was collected by centrifugation (1580 MGR, Gyrozen, Daejeon, Korea) at 10,000× *g* for 20 min and washed twice with cold sterile water. The bacterial pellet was finally freeze-dried in a deep freezer (−80 °C) before further experiments. The lyophillized PA and PR strains were dissolved in distilled water at a final concentration of 1.25 × 10^9^ cfu/mL before use.

### 2.2. Animals and Diets

Forty C57BL/6J male mice were purchased from Central Lab. Animal Inc. (Seoul, Korea) at 4 weeks of age. The mice had free access to water and were adapted to commercial pelleted feed (Research Diets, New Brunswick, NJ, USA) for 1 week. They were then separated into the following four groups using the randomized block design method: ND, normal diet group; HD, high fat diet group; HD-PR, HD plus 4 mL/kg body weight reference stain group; HD-PA, HD plus 4 mL/kg body weight *P*. *acidilactici* M76. All diets were obtained from Research Diets, Inc. The ND group received the normal diet (D12450B) with 10% kcal% fat (3.85 kcal/g), whereas the three treatment groups (HD, HD-PR, and HD-PA) were provided the high fat diet (D12492) with 60% kcal% fat (5.24 kcal/g).

The lyophilized samples of PR and PA were cultured and tested for purity using 16S rRNA sequencing technique [19]. The samples were contamination free and were then used for the experiments. The lyophilized PA and PR strains were given orally to animals at a dose of 4 mL/kg body weight for 12 weeks. The animals were housed under a 12 h light and 12 h dark cycle and given free access to food and water during the entire experimental period. Food intake and body weight were measured daily and weekly, respectively. The experimental protocol was approved by the Animal Care and Use Committee of Chonbuk National University (CBU 2012-0041, 13 September 2012).

### 2.3. Animal Treatment and Biochemical Assays

Blood samples were collected after a 12 h overnight fast and kept on ice for 1 h. Serum was separated from the blood by centrifugation at 1100× *g* for 15 min at 4 °C (Micro 17R; Hanil Science In Co., Ltd., Gangneung, Korea) and stored at −70 °C until analyses. After blood collection, organs such as liver and epididymal fat were surgically removed, washed in phosphate-buffered saline solution, wiped with a paper towel, and stored at −70 °C until analyses.

Serum triglyceride (TG), total cholesterol (TC) concentration, aspartate aminotransferase (AST) and alanine aminotransferase (ALT) activities were measured using a DRI-CHEM 3500i machine (Fujifilm, Tokyo, Japan). TG and TC concentrations in liver tissue were measured by an enzymatic method using a commercial kit (Asan Pharmaceutical Co., Seoul, Korea).

### 2.4. Analysis of Insulin and Leptin

Serum insulin and leptin concentrations were measured by enzyme-linked immunosorbent assay using a commercial Mouse Insulin kit (Shibayagi, Shibukawa, Japan) and Quantikine^®^ Immunoassay kit (R & D System, Minneapolis, MN, USA), respectively.

### 2.5. Hepatic mRNA Expression Analysis of Lipid-Regulating Genes

Total RNA was extracted from liver tissue using Trizol reagent (Invitrogen Life Technologies, Carlsbad, CA, USA), and concentrations were measured spectrophotometrically. The extracted RNA was reverse transcribed into complementary DNA using a high capacity cDNA reverse transcription kit (Applied Biosystems, Foster City, CA, USA). Then, RNA expression level was quantified by a quantitative real-time polymerase chain reaction (PCR) using SYBR Green PCR Master Mix (Applied Biosystems, Woolston, Warrington, UK) and the 7500 Real-Time PCR system (Applied Biosystems, USA) according to the manufacturer’s protocol. The gene specific primers used are given in Table 1. Relative quantification of gene expression with the real-time PCR data was calculated relative to that of the β-actin.

**Table 1:** Sequences of primers for mRNA gene analysis in liver.

	Primer	Sequence
ACC	Forward	5′-CCA ACA TGA GGA CTA TAA CTT CCT-3′
	Reverse	5′-TAC ATA CGT GCC GTC AGG CTT CAC-3′
FAS	Forward	5′-AGG GGT CGA CCT GGT CCT CA-3′
	Reverse	5′-GCC ATG CCC AGA GGG TGG TT-3′
PPARγ	Forward	5′-GCT GTT ATG GGT GAA ACT CTG-3′
	Reverse	5′-ATA AGG TGG AGA TGC AGG TTC-3′
CPT-1	Forward	5′-AAA GAT CAA TCG GAC CCT AGA CA-3′
	Reverse	5′-CAG CGA GTA GCG CAT AGT CA-3′
β-actin	Forward	5′-GTG GGG CGC CCC AGG CAC CAG-3′
	Reverse	5′-CTC CTT AAT GTC ACG CAC GAT TTC-3′

### 2.6. Statistical Analyses

All values are expressed as mean ± SEM. Data was analyzed with one-way ANOVA using SPSS version 12.0 (SPSS Inc., Chicago, IL, USA). Differences among groups were assessed using Duncan’s multiple range test. A *p*-value <0.05 was considered significant.

## 3. Results

### 3.1. Body Weight, Epididymal Fat Pad Weight, Food Intake, and Energy Intake

No significant differences were observed in initial body weight. C57BL/6J mice fed with high fat diet for 12 weeks became obese associated with both increased body weight and epididymal fat. However, by the end of the study, body weight and epididymal fat in the PA group were significantly lower than those in the HD group (Table 2). Feed and energy intake was also reduced in the PA group compared with that in the HD group.

**Table 2:** Body weight, epididymal fat, food intake and energy intake.

Groups	ND	HD	HD-PR	HD-PA
Initial body weight (g)	18.86 ± 0.77 ^NS^	19.00 ± 0.43	19.05 ± 1.43	19.13 ± 0.68
Final body weight (g)	23.72 ± 1.07 ^c^	40.69 ± 3.58 ^a^	39.37 ± 2.60 ^ab^	37.63 ± 4.38 ^b^
Epididymal fat (g)	0.43 ± 0.19 ^c^	3.02 ± 0.82 ^a^	2.53 ± 0.48 ^ab^	2.26 ± 0.58 ^b^
Feed intake (g/day)	2.06 ± 0.05 ^c^	2.43 ± 0.18 ^a^	2.19 ± 0.07 ^bc^	2.32 ± 0.11 ^ab^
Energy intake (kcal/day)	7.92 ± 0.21 ^c^	12.71 ± 0.93 ^a^	11.45 ± 0.36 ^b^	12.14 ± 0.55 ^ab^

Values are expressed as the mean ± SEM, *n =* 10 mice per group. Values with different superscript alphabets in the same row are significantly different (*p* < 0.05) as assessed by one-way ANOVA and the Duncan’s multiple range test. NS, not significant. ND, normal diet (D12450B) with 10% kcal% fat (3.85 kcal/g); HD, high fat diet (D12492) with 60% kcal% fat (5.24 kcal/g); HD-PR, high-fat diet plus *P*. *acidilactici* DSM 20,284 reference strain 4 mL/kg body weight; HD-PA, high-fat diet plus *P*. *acidilactici* M76 strain 4 mL/kg body weight.

### 3.2. Serum and Hepatic Lipid Profiles

Serum and hepatic lipid profiles are shown in Table 3. Serum TC decreased significantly in the HD-PA group compared with that in the HD group. Serum TG was not significantly different among the groups. However, hepatic TC and TG decreased significantly in the HD-PA group compared with those in the HD group.

**Table 3:** Lipid concentration in serum and liver.

	Groups			
	ND	HD	HD-PR	HD-PA
*Serum (mg/dl)*				
Total cholesterol	109.80 ± 8.51 ^c^	212.40 ± 22.60 ^a^	207.00 ± 22.51 ^a^	187.40 ± 16.60 ^b^
Triglyceride	110.80 ± 46.60 ^NS^	130.20 ± 53.66	121.40 ± 49.08	140.00 ± 58.80
*Liver (mg/g)*				
Total cholesterol	3.82 ± 2.15 ^c^	12.35 ± 4.83 ^a^	10.51 ± 5.18 ^ab^	8.59 ± 4.93 ^b^
Triglyceride	48.31 ± 12.45 ^c^	99.49 ± 36.16 ^a^	88.73 ± 29.49 ^ab^	79.94 ± 24.66 ^b^

Values are expressed as the mean ± SEM, *n =* 10 mice per group. Values with different superscript alphabets in the same row are significantly different (*p* < 0.05) as assessed by one-way ANOVA and the Duncan’s multiple range test.

### 3.3. Serum Leptin and Insulin Concentrations

**Figure 1:**
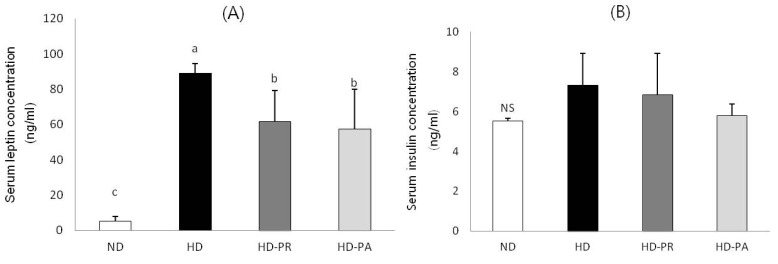
(**A**) Serum leptin and (**B**) insulin levels. Bars represent the mean ± SEM, *n =* 10 mice per group. Values with different superscripts are significantly different (*p* < 0.05) as assessed by one-way ANOVA and the Duncan’s multiple range test.

Serum leptin and insulin levels are shown in Figure 1. Serum leptin level was significantly lower in the HD-PA and HD-PR groups than those in the HD group. Serum insulin levels were measured to examine the changes in insulin level. The level of serum insulin in HD-PA group was at a similar level to ND group.

### 3.4. Hepatic mRNA Level

**Figure 2:**
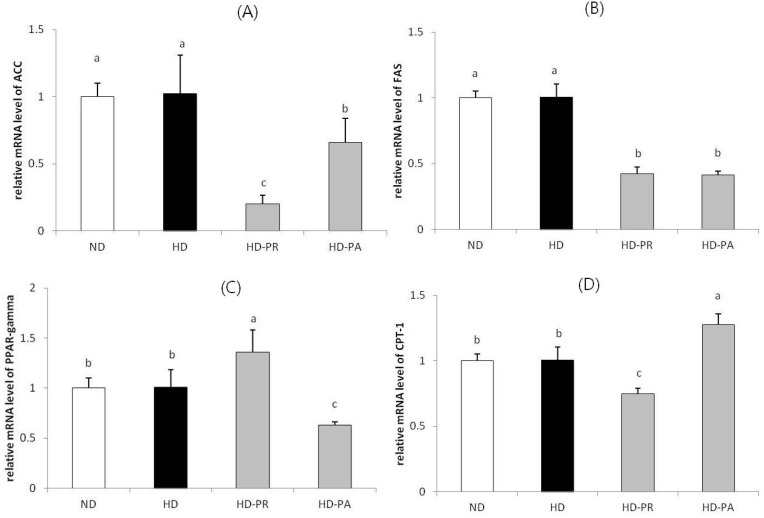
Hepatic mRNA expression of (**A**) ACC; (**B**) FAS; (**C**) PPAR-γ and (**D**) CPT-1. Bars represent the mean ± SEM, *n =* 10 mice per group. The mRNA levels of experimental groups were normalized to the level of the normal diet group. Values with different superscripts are significantly different (*p* < 0.05) as assessed by one-way ANOVA and the Duncan′s multiple range test.

Relative mRNA levels of genes (Figure 2) involved in hepatic lipid metabolism, including acetyl-CoA carboxylase (ACC), fatty acid synthase (FAS), peroxisome proliferator-activated receptor gamma (PPAR-γ), and carnitine palmitoyltransferase-1 (CPT-1) were measured in liver tissues. The level of these genes were at a similar range in high fat diet fed HD group as that of the normal diet fed ND group. However, the ACC and FAS mRNA were at significantly low levels in the HD-PA and HD-PR groups compared to those in the HD group. The PPAR-γ mRNA level in the HD-PA group was significantly lower than that in the HD group. CPT-1 mRNA was higher in the HD-PA group than that in the HD group.

### 3.5. Hepatic Toxicity Assay

**Figure 3:**
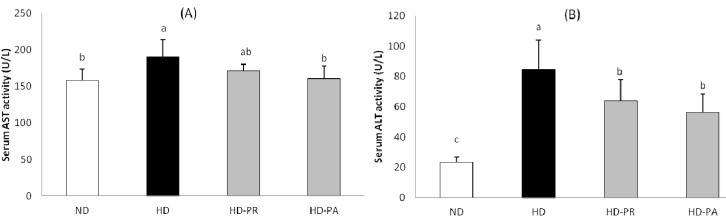
(**A**) Serum aspartate aminotransferase and (**B**) alanine aminotransferase levels. Bars represent the mean ± SEM, *n =* 5 mice per group. Values with different superscripts are significantly different (*p* < 0.05) as assessed by one-way ANOVA and the Duncan’s multiple range test.

The serum aspartate aminotransferase (AST) and alanine aminotransferase (ALT) are shown in Figure 3. The serum AST level was higher in the HD group, while the PA supplemented group showed a lower level of AST comparing to the HD group, which was at a similar range as that of the ND group. Similarly the serum ALT level in the high fat diet fed subgroups was higher than the normal diet fed ND group. However the PA and PR supplemented group showed significantly lower level of serum ALT than that of the HD group.

## 4. Discussion

LAB are the most common and dominant microorganisms present in fermented foods. The increased health awareness among people and recent studies support the role of LAB as probiotics [21]. In high fat diet-induced obese rodent model, the anti-obesity effects of *Pediococcus* sp. [22], *Lactobacillus* sp. [13,23,24], and *Bifidobacterium* sp. [25,26] have been reported by several investigators. For example, An *et al*. reported that administration of LAB reduced body and fat weights, blood serum levels and significantly increased fecal LAB counts [25].

We examined the effects of *P*. *acidilactici* M76 on lipid metabolism in C57BL/6J mice by assessing lipid profiles and alterations in mRNA levels of enzymes related to synthesis and degradation of lipids. High fat diet-induced obesity is associated with adipocyte hypertrophy and leptin resistance [27]. Intake of dietary fat promotes the development of obesity in C57BL/6J mice [28]. Our results support previous studies showing that a high fat diet induces higher body fat deposition and increases bodyweight [29]. Our results revealed that final body weight and epididymal fat in the high fat diet groups were significantly higher than those in the ND group; however, animals in the HD-PA group had lower final body weight and epididymal fat compared to those in the HD group (Table 2). Interestingly, feed intake (*p =* 0.066) and energy intake (*p =* 0.069) was not significantly different among the high fat diet fed HD, HD-PR, and HD-PA groups.

*Lactobacillus plantarum* PL60 reduced the weights of epididymal, inguinal, mesenteric, and perirenal white adipose tissues and significantly reduced the blood levels of total glucose and body weights of mice [30]. Zhao *et al*. demonstrated that *P*. *pentosaceus* reduces body weight gain and liver lipid contents (TG and TC) in mice fed a high fat diet for 8 weeks [22]. 

Intake of LAB and prebiotics reported to affect the cholesterol metabolism [31] while LAB alone significantly promotes the hypocholesterolemic effect *in vivo* [32,33,34,35]. Further, the presence of exopolysaccharide (EPS) upsurge the hypocholesterolemic effect of LAB in ingested animals [36,37]. Similarly, in our study supplementation of EPS producing lactic acid bacteria *P*. *adicilactici* M76 (PA) strain lowers the high fat diet-induced elevation of TC in both serum and liver. A lower level of hepatic TG was observed in the PA supplemented group (Table 3). The serum triglyceride was higher in the normal diet fed ND group which was very close to the high fat diet fed HD group. Dietary sucrose intake has been observed to be correlated with raise in serum triglyceride in animals [38]. The normal diet composed of high amount of sucrose, that might induced the raise in serum triglyceride in the ND group. However in the high fat diet group continuous supplementation with the PA strain for 12 weeks improved serum and hepatic lipid profiles and also decrease the raise in serum and hepatic TC level compared to the HD and PR group. These results indicate that PA was more effective for decreasing visceral and hepatic fat accumulation and preventing hepatic TG than those in mice fed the reference strain (PR). With increase in hepatic lipid accumulation, the level of serum AST and ALT raises [39]. In our study the animal fed with high fat diet showed elevated level of AST and ALT comparing with the normal diet fed group; however the animals treated with PA and PR showed lower level of the enzymes when compared with the HD group (Figure 3). The low level of serum AST and ALT were similar as of the hepatic triglyceride level in the respective groups indicating that PA and PR treatment prevented the high fat diet mediated liver damage in these treated animals.

We reported previously that the *Pediococcus acidilactici* M76 (PA) strain isolated from makgeolli produces a functionally active EPS. The purified EPS shows a proliferative effect on the pancreatic RIN-m5F cell line and remarkable protective activity against alloxan-induced cytotoxicity [19]. To exert their probiotic effects, maintenance of LAB in the gastrointestinal tract is necessary to prevent their rapid removal by contraction of the gut. The ability to adhere to mucosa is required for long-term colonization and persistence in the gut [40]. Therefore, further research, including human studies, to elucidate the mechanisms of action and the relative influence of acid/bile tolerance and adhesive properties is needed.

Adipose tissue produces the hormone leptin, which is released into the blood stream. As fat deposits increase, blood leptin levels also tend to increase. Thus, leptin levels are closely related to the percentage of body fat, and markedly higher serum leptin levels are found in obese individuals compared with non-obese individuals [41]. Many studies have reported [25,42] that a continuous high fat diet results in increased serum leptin. In our study, serum leptin concentration decreased significantly in the HD-PA group compared with that in the HD group; showing congruency with other studies using LAB on C57BL/6J mice fed a high fat diet [43]. For example, a high fat feeding group showed a 9-fold increase in plasma leptin compared with that in the normal diet fed group. After treatment with *Lactobacillus plantarum*, circulating leptin levels decreased significantly (two fold). Results of these studies suggest that reductions in fat mass and body weight are associated with a reduction in leptin. Although many studies elucidated the insulin lowering effects of LAB, however, we were not able to observe any significant difference in the level of serum insulin among the the high fat diet fed groups (Figure 1). This could be due to insufficient quantity of PA and PR supplementation to exert the insulin lowering effects in these animals. Also, a high level of serum insulin was observed in normal diet fed ND group which was in similar range as that of the high fat diet fed group. Some studies demonstrated that intake of dietary sucrose raises serum insulin level [44,45]. The ND diet contained 350 g/kg of sucrose that could be the reason for raise of insulin (5 ng/mL) in the ND groups.

Inhibiting ACC, with the resulting inhibition of fatty acid synthesis and stimulation of fatty acid oxidation, has the potential to favorably affect the multitude of cardiovascular risk factors associated with metabolic syndrome [46]. ACC catalyzes the synthesis of malonyl-CoA, a key rate-controlling step in fatty acid oxidation, which acts as an intermediate in fatty acid synthesis and an allosteric inhibitor of CPT-1. CPT-1 regulates the transfer of long-chain acyl-CoAs from the cytosol to the mitochondria where they are oxidized [47]. In this study, the hepatic ACC and FAS levels of the animals in the ND and HD group were at a similar range, this could be due to high carbohydrate content in the diet of ND group, that might accelerated the synthesis of the ACC and FAS mRNA resulting in a similar level of these genes in comparison with the HD group. However, the ACC and FAS hepatic mRNA levels decreased significantly in the HD-PA group compared to those in the HD group, whereas CPT-1 genes involved in mitochondrial fat oxidation increased significantly in the HD-PA group compared to those in the HD group (Figure 2). Yoo *et al*. showed that hepatic FAS enzyme and mRNA levels decrease as a result of administration of *L*. *plantarum and L*. *curvatus* in C57BL/6J mice fed a high fat diet [48]. PPAR-γ stimulates lipolysis of circulating TGs and subsequent uptake of fatty acids into adipose cells. It also stimulates binding and activation of fatty acids in the cytosol, which is required for TG synthesis [49]. As shown in our results, FAS, ACC, and PPAR-γ levels were markedly suppressed in the HD-PA group, whereas CPT-1 level was markedly enhanced in the HD-PA group compared with that in the HD group. Therefore, we suggest that the reduction of hepatic TG levels in the HD-PA group was attributable to the suppression of fatty acid synthesis and enhanced fatty acid β-oxidation.

In non-adipocytes (islets) of leptin-resistant ZDF (*fa/fa*) rats, TG accumulation is greater than that in normal islets. This difference is attributed to high expression of fatty acid synthesis enzymes (ACC and FAS) and of their transcription factor PPAR-γ coupled with reduced expression of fatty acid oxidation enzymes such as acyl CoA oxidase, and CPT-1 [50,51]. We also found decreased serum leptin and hepatic TGs, increased levels of CPT-1, and decreased levels of ACC, FAS, and PPAR-γ in the PA strain supplemented group. These data correlated with observable changes in body weight in the HD-PA group compared to those in the HD group.

In summary, our results show that a 12 week oral treatment with PA isolated from traditional makgeolli was beneficial for reducing fat pad mass and preventing increase of body weight. The PA strain was effective in lowering serum TC in animals along with averting the raise of serum leptin. The treatment with PA also modulated the expression of hepatic lipid metabolic genes like PPAR-γ, FAS, and ACC gene expression. The PA strain efficiently reduced the hepatic TGs and TC, and the response was associated with increased CPT-1, which is involved in the catabolism of lipids via beta-oxidation.

## 5. Conclusions

These results suggest that the *Pediococcus acidilactici* M76 has lipid lowering effects in high fat diet induced obese mice.
